# The Effectiveness of Medical Adherence Mobile Health Solutions for Individuals With Epilepsy: Protocol for a Systematic Review

**DOI:** 10.2196/55123

**Published:** 2024-08-06

**Authors:** Pantea Keikhosrokiani, Manria Polus, Sharon Guardado Medina, Minna Isomursu

**Affiliations:** 1 Faculty of Information Technology and Electrical Engineering University of Oulu Oulu Finland; 2 Faculty of Medicine University of Oulu Oulu Finland

**Keywords:** digital care pathway, epilepsy, mHealth, mobile health, effectiveness, systematic review, management, medical adherence, patient outcomes, digital health, design, eHealth solutions, health care professionals

## Abstract

**Background:**

Epilepsy requires continuous management and treatment to optimize patient outcomes. The advancement of digital health has led to the development of various mobile health (mHealth) tools designed to enhance treatment adherence among individuals with epilepsy. These solutions offer crucial support through features such as reminders, educational resources, personalized feedback, assistance with managing costs, shared decision-making, and access to supportive communities. To design effective medication adherence mHealth solutions, it is essential to evaluate the effectiveness of existing mHealth tools, understand the unique circumstances of different patients, and identify the roles of health care professionals within the digital care pathway. Existing studies on epilepsy primarily focus on self-management, whereas the effectiveness and usability of medical adherence mHealth solutions often remain overlooked. Furthermore, the involvement of health care professionals in digital care pathways for epilepsy as well as the impact of adherence mHealth solutions on the patient experience have not been adequately explored.

**Objective:**

This study aims to assess the effectiveness of current mHealth solutions designed to improve medical adherence among patients with epilepsy. Furthermore, the study will examine the experiences of patients using mHealth solutions for maintaining medical adherence in epilepsy care. Finally, this review intends to determine the roles of health care professionals within mHealth systems aimed at supporting adherence to medication among patients with epilepsy.

**Methods:**

A systematic literature review has been selected as the appropriate method to address the research questions, adhering to the PRISMA (Preferred Reporting Items for Systematic Reviews and Meta-Analyses) guidelines. The inclusion and exclusion criteria have been carefully selected, and both qualitative and quantitative analyses will be used to analyze the results. The expected results will mainly focus on the comparison, classification, and analysis of the effectiveness of current medical adherence mHealth tools. Moreover, the patient experiences using available medical adherence mHealth tools for epilepsy will be assessed. Finally, the role of health care professionals in the epilepsy digital care pathway will be explored, with emphasis on medical adherence.

**Results:**

The initial search, full-text screening, and data extraction have been carried out. Thirty-three papers were included in the final stage of the review. The study is expected to be completed by October 2024.

**Conclusions:**

To enhance the digital care pathway for epilepsy, a medical adherence mHealth solution should be personalized, manage medications, include an alarm system, track seizures, support consultations, and offer updated treatment plans. This study aims to understand how findings from the research questions can improve mHealth solutions for individuals with epilepsy. Insights from this research on the effectiveness of current mHealth adherence solutions will provide guidance for developing future mHealth systems, making them more efficient and effective in managing epilepsy.

**Trial Registration:**

PROSPERO CRD4202347400; https://tinyurl.com/48mfx22e

**International Registered Report Identifier (IRRID):**

DERR1-10.2196/55123

## Introduction

Epilepsy is considered a chronic neurological condition that has to be consistently managed and treated to optimize patient outcomes [1]. With the advancement of digital health technologies, various mobile health (mHealth) solutions have been developed to improve the adherence of patients with epilepsy to treatment [2]. Medication adherence mHealth solutions can offer significant assistance to individuals with epilepsy, including reminders and alerts, education, personalized feedback, cost management, support, and community engagement [3-6].

When designing medication adherence mHealth solutions, it is crucial to understand the unique circumstances of different patients. Patients can have many potential reasons for nonadherence. Previous research identified common barriers for medication adherence for children with epilepsy, including disliking the taste of the medication, difficulty remembering to give epilepsy medications up to 4 times per day, lower family support, and lower family socioeconomic status [1,2]. For adults, common reasons for nonadherence include the beliefs about medications, being depressed or anxious, poor self-management, uncontrolled seizures, demanding medication routine, poor relationship with physician, and perceived social support [2,4,6]. Some patients may also find digital systems inaccessible or difficult to use [5]. Understanding the diverse needs, preferences, and barriers of patients is crucial, as these factors can impact the effectiveness of mHealth interventions. Similarly, previous studies have identified that patients frequently report a perceived lack of communication with their health care professionals as a barrier to understanding their health conditions and using available tools for epilepsy self-management [7].

Reviewing studies related to digital health solutions for epilepsy is essential for the determination of effective interventions and how they could be leveraged beyond individual use in the health care context. Existing reviews on this topic [7-10] have mainly focused on overviewing the digital tools for epilepsy and self-management; however, mHealth solutions specifically designed for medical adherence were neglected. Accordingly, data on the usability, acceptance, and effectiveness of these mHealth solutions remain scattered across individual studies.

Previous studies have shown that health care professionals in epilepsy care are using smartphone apps with their patients, and they largely perceive that data from wearables might be useful to enrich their understanding of a patient’s health state in between consultations and for improving adherence to medications; however, further analysis showed that the data generated by these solutions were rarely or never used or discussed in the clinic during consultations [11]. This suggests that the role of health care professionals in mHealth interventions for epileptic care has not been properly addressed. The active participation of health care professionals is required for a digital care pathway to optimize the medical adherence of patients to mHealth solutions. Furthermore, the existing reviews [6,12] have not thoroughly assessed the patient’s experience of using digital solutions for medical adherence in epilepsy. Therefore, a thorough synthesis of the body of research assessing the benefits and drawbacks of mHealth adherence solutions for individuals with epilepsy is required.

In this regard, this systematic review aims to collate, synthesize, and summarize the existing studies to provide a clear understanding of the current state of mHealth solutions for treatment adherence in epilepsy and to identify areas for future research. Based on the limitations and shortcomings of existing studies on the topic, the following 3 research questions were formulated to be answered in this systematic review:

1. What are the effects of medical adherence mHealth solutions on individuals with epilepsy in managing their condition?
2. What are the effects of medical adherence mHealth solutions on the patient’s experience? 
3. What are the effects of health care professionals on the medical adherence of individuals with epilepsy using mHealth solutions?

The overall goal of this study is to systematically review the existing research related to mHealth solutions for medical adherence among individuals with epilepsy. To address the research questions, this systematic review aims to address 3 main objectives. First, it explores the effectiveness of various medical adherence mHealth solutions for individuals with epilepsy, assessing how these technologies assist in managing their condition. Second, the review investigates the experiences of patients using these mHealth solutions, focusing on how the technology impacts their adherence behaviors and overall treatment engagement. Lastly, the study aims to identify the roles and responsibilities that health care professionals are expected to assume within the context of mHealth solutions designed to improve medical adherence for epilepsy. This comprehensive approach can help to illuminate the multifaceted impacts of mHealth technologies in the management of epilepsy. This review can consequently provide insights for improving the quality of medical adherence mHealth solutions for individuals with epilepsy. Therefore, this study represents a critical stepping stone for future research on mHealth solutions for individuals with epilepsy.

## Methods

### Study Objectives and Design

**Figure 1:**
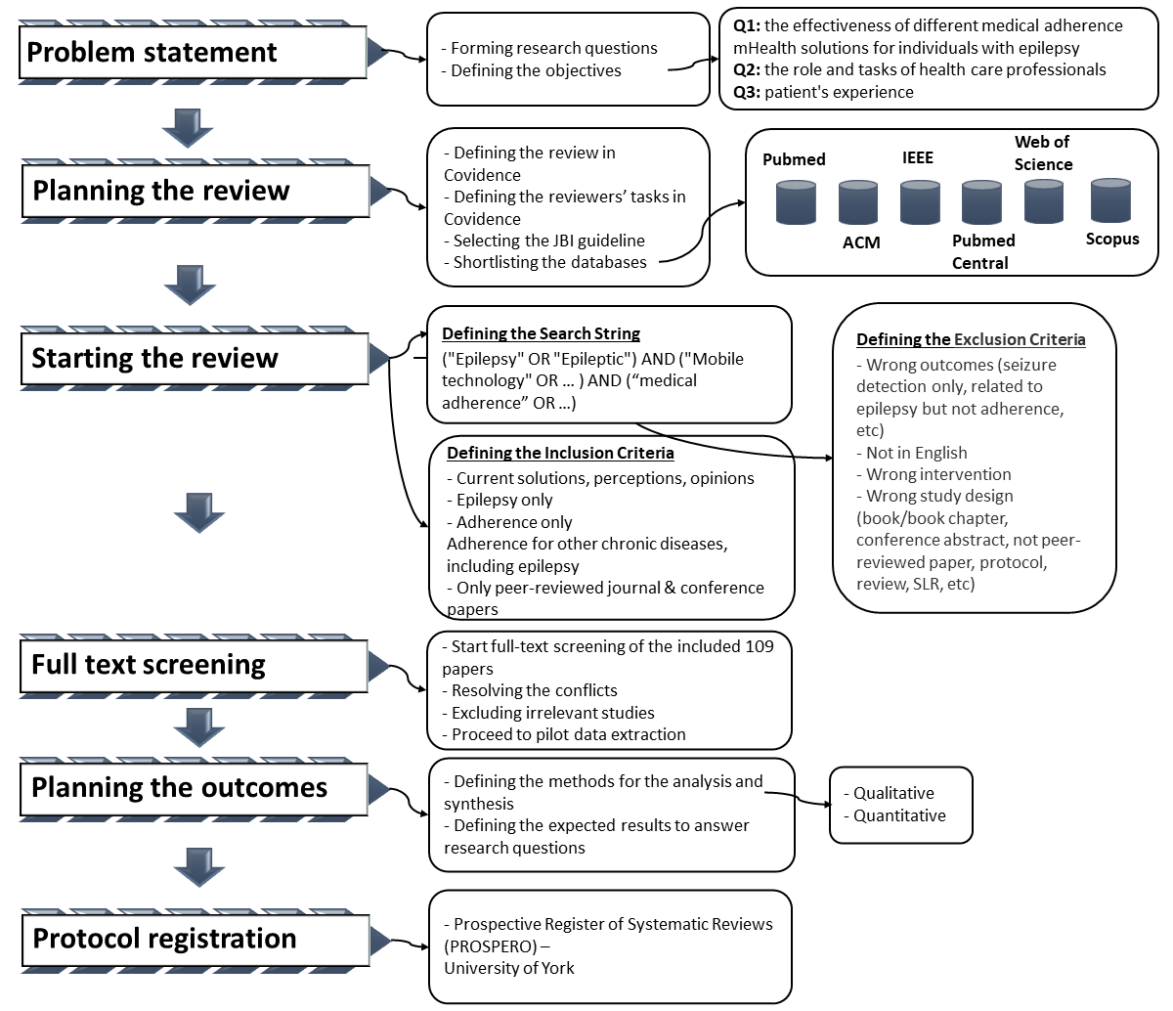
The protocol process flow. ACM: Association for Computing Machinery; IEEE: Institute of Electrical and Electronics Engineers; JBI: Joanna Briggs Institute; mHealth: mobile health; SLR: systematic literature review.

This study aims to provide a comprehensive overview of the available evidence and prior research on medical adherence mHealth solutions for individuals with epilepsy. For this reason, a systematic literature review (SLR) was selected to answer the research questions. The SLR will be carried out according to the PRISMA (Preferred Reporting Items for Systematic Reviews and Meta-Analyses) statement, which provides a reliable methodology to perform SLRs in the fields of medicine. The process flow of the protocol is illustrated in Figure 1.

The first step involves the formulation of the 3 research questions based on the shortcomings of existing studies. The review is then planned to be carried out using Covidence according to the Joanna Briggs Institute (JBI) guideline for a systematic review of effectiveness evidence [13]. In the next step, the search string along with inclusion and exclusion criteria are defined. Subsequently, the full-text screening step is performed for the included papers. Each paper will be reviewed by at least 2 reviewers and conflicts will be resolved through discussion and using Covidence. After finalizing the included full papers, the analysis and synthesis are carried out along with interpretation of the results.

### Information Sources and Search Strategy

Extensive searches were carried out for relevant literature in digital databases specializing in the fields of information technology, biomedical engineering, and health to find the eligible studies, including PubMed, ACM Digital Library, IEEE Xplore, PubMed Central, Web of Science, and Scopus. The main search string used for searching in the digital databases includes terms related to epilepsy, digital interventions, and medical adherence, including the Boolean operators “AND” and “OR,” as follows: (“Epilepsy” OR “Epileptic”) AND (“Mobile technology” OR “Mobile technologies” OR “mobile phone” OR “mobile device” OR “mobile phones” OR “mobile devices” OR “smart device” OR “smartphone” OR “smartwatch” OR “smart ring” OR “smart device” OR “smart devices” OR “mobile app” OR “health app” OR “mobile health intervention” OR “mobile health solution” OR “mHealth” OR “mHealth” OR “mobile health tool”) AND (“medical adherence” OR “adherence” OR “medication” OR “adherence to medicines” OR “medicine adherence” OR “medicine compliance” OR “medical advice” OR “adhering to medical advice” OR “adherence to medical advice” OR “treatment”). The corresponding Medical Subject Headings (MeSH) terms and keywords were combined for the search strategy, especially in PubMed.

### Selection of Studies and Eligibility Criteria

This systematic review will include original research articles focused on current medical adherence mHealth solutions for individuals with epilepsy. The eligible publications for this review are restricted to those published in peer-reviewed journals and conference proceedings written in English. The publication period of the included studies was restricted from 2013 to 2023. Although mHealth technologies started to become more accessible in the first half of the 2010s, their impact on the health care scene started to become evident only several years later. In 2013, it was acknowledged that only a few studies had assessed the impact of mobile apps in the health context, and all of those studies referred to apps that had been created only for research purposes and were not available to the public at that time [14]. Therefore, we limited our search to articles with publication dates starting only after 2013. Furthermore, articles based on perceptions, opinions, and futuristic solutions, especially those focusing on medical adherence for epilepsy, will be reviewed for this study. The methods used for data collection are varied, including qualitative, quantitative, and mixed methods approaches. Review papers, SLRs, and abstracts are excluded from study selection. Moreover, articles that are focused on a technical approach of epileptic seizure detection rather than on mHealth solutions as an intervention for epilepsy management are excluded from the review.

The included articles will be imported into Covidence (Veritas Health Innovation). The screening process is divided into two stages, which are carried out independently by at least 2 researchers with computer science and health psychology backgrounds along with previous research experience in the field. At the initial stage, the screening is limited to titles and abstracts. Before starting this stage, the reviewers complete a joint exercise to validate the methodology and ensure the inclusion and exclusion criteria were correctly understood. Any disagreements that arise during the initial screening stage are discussed and resolved between the 2 reviewers before starting the second stage. The second screening stage includes the review of the full text of the articles that are preliminarily included from the initial stage.

### Risk of Bias and Quality Assessment

Assessing the quality of reviewed literature and the risk of bias is necessary to ensure the reliability of any conclusions made for an SLR [1]. This study will follow the quality assessment guideline provided by Yang et al [1]. In addition, the quality assessment checklist designed by Dybå and Dingsøyr [15] will be used to evaluate the risk of bias and the quality of the studies included in the data extraction process.

### Data Extraction and Analysis

A data extraction form created in Covidence will be used to gather and extract the relevant information from the included articles. The study design will be classified based on the tool from Grimes and Shulz [16]. Three reviewers will work on the data extraction task.

A mixed methods approach will be used to analyze the included studies to determine the overall evidence of the effectiveness of mHealth interventions for individuals with epilepsy. To answer the first research question, the effectiveness of mHealth interventions will be assessed using the guideline provided by the JBI [13] based on both qualitative and quantitative analyses. Furthermore, using quantitative analysis, the efficacy of the mHealth solutions will be evaluated and classified according to various features and phases.

The second research question will be addressed through qualitative studies regarding the experiences of patients and their caregivers with using mHealth interventions for medical adherence. The qualitative data will provide insights on patients’ needs, preferences, challenges, and barriers of using mHealth interventions. The information about patients’ experiences will complement the quantitative results and help us to better understand the results regarding the effectiveness of the mHealth interventions.

To maximize the benefits of mHealth solutions for medical adherence, health care providers must actively participate in the digital care pathway. Hence, the last research question will be addressed through qualitative analysis to explore the effectiveness, role, and responsibilities of health care professionals on medical adherence mHealth solutions for individuals with epilepsy.

Specifically, quantitative data such as year of publication and number of adherence criteria will be analyzed. Descriptive analysis and thematic synthesis will also be used to describe the effectiveness of the mHealth solutions. A coding strategy will be applied using NVivo software to simplify the synthesis of qualitative data. Furthermore, textual analytics will be used for clustering purposes. The effectiveness of the mHealth solutions will be categorized and grouped based on different characteristics and stages using quantitative analysis, while different categories will be labeled and classified quantitatively.

## Results

The protocol was registered in the International Prospective Register of Systematic Reviews (PROSPERO; University of York) under registration number CRD42023474008. As of July 2024, the initial searches have been carried out, the manuscripts have been imported into Covidence, and the full-text screening process has begun. Based on the initial search in the electronic databases, 4873 relevant articles published from 2013 to 2023 were identified. After applying the exclusion criteria; filtering; and removing review papers, SLRs, abstracts, and technical papers related to seizure detection, 477 papers were imported into Covidence. A total of 120 duplicates were removed from Covidence. In the first screening stage, the titles and abstracts of 357 articles were screened. From the initial screening, 109 papers were selected for full-text screening as they fulfilled the inclusion criteria. After full-text screening, a total of 33 papers were included in this review. This systematic review is anticipated to be completed by the end of 2024.

## Discussion

To enhance the current epilepsy digital care pathway, a medical adherence mHealth solution is needed that personalizes the care plan, manages medication, sets an alarm system, monitors and tracks seizures, facilitates consultations, and provides updated treatment plans. Therefore, this study aims to determine the effectiveness of various medical adherence mHealth solutions for individuals with epilepsy. By addressing this aspect, effective mHealth solutions can improve the quality of care, enhance treatment adherence, and consequently yield better health outcomes. Patient experience plays a crucial role in determining effective medical adherence through mHealth solutions for individuals with epilepsy, as it leads to treatment plans and the overall management of the digital care pathway. Furthermore, clearly defining the role and effect of health care professionals is crucial in enhancing medical adherence to mHealth solutions for epilepsy. Health care professionals can significantly affect patient engagement, trust, and adherence to the treatment plan.

Several key points can be addressed by this study to elaborate on the findings, integrate them with existing knowledge, and outline future research directions. First, the results of this systematic review will confirm, extend, or challenge existing knowledge about mHealth solutions for epilepsy. The effectiveness of various mHealth solutions in enhancing medical adherence among patients with epilepsy will be interpreted, highlighting any significant trends or unexpected outcomes observed in the literature. Furthermore, the results will provide a detailed analysis of patient experiences with mHealth technologies and discuss factors that influence patient engagement and adherence to treatment, such as usability, accessibility, and personalized features of mHealth solutions. Finally, the findings of this study can provide new insight into the evolving roles and responsibilities of health care professionals in deploying and managing mHealth solutions, while highlighting the training needs, challenges, and opportunities for health care providers to effectively integrate mHealth into a digital care pathway for epilepsy.

After addressing the research questions, this study will explore how insights derived from the research findings can inform improvements in the design of medical adherence mHealth solutions for individuals with epilepsy. Drawing from the critical insights obtained regarding the effectiveness of existing solutions, future mHealth systems can be refined to provide a more efficient and sustainable digital care pathway for individuals with epilepsy [17,18]. Finally, the outcomes of this study are expected to encourage collaborative, patient-centered care that may improve adherence to the same degree as other digital tools. However, accurate measurement of medical adherence and the effectiveness classification might be challenging, which are identified as the main limitations of this study.

## Abbreviations

JBI: Joanna Briggs Institute
MeSH: Medical Subject Headings
mHealth: mobile health
PRISMA: Preferred Reporting Items for Systematic Reviews and Meta-Analyses
PROSPERO: International Prospective Register of Systematic Reviews
SLR: systematic literature review
